# A non-mosaic *PORCN* mutation in a male with severe congenital anomalies overlapping focal dermal hypoplasia

**DOI:** 10.1016/j.ymgmr.2017.06.002

**Published:** 2017-06-07

**Authors:** Simran Madan, Wei Liu, James T. Lu, V. Reid Sutton, Bryant Toth, Priscilla Joe, John R. Waterson, Richard A. Gibbs, Ignatia B. Van den Veyver, Edward J. Lammer, Philippe M. Campeau, Brendan H. Lee

**Affiliations:** a Department of Molecular and Human Genetics, Baylor College of Medicine, Houston, TX, USA; b Interdepartmental Program in Translational Biology and Molecular Medicine, Baylor College of Medicine, Houston, TX, USA; c Department of Obstetrics and Gynecology, Baylor College of Medicine, Houston, TX, USA; d Human Genome Sequencing Center, Baylor College of Medicine, Houston, TX, USA; e Department of Structural and Computational Biology & Molecular Biophysics, Baylor College of Medicine, Houston, TX, USA; f Children's Hospital & Research Center, Oakland, CA, USA; g Howard Hughes Medical Institute, Houston, TX, USA

**Keywords:** Focal dermal hypoplasia, Goltz syndrome, PORCN, Non-mosaic, Micro-opthalmia

## Abstract

Mutations in the *PORCN* gene cause the X-linked dominant condition focal dermal hypoplasia (FDH). Features of FDH include striated pigmentation of the skin, ocular and skeletal malformations. FDH is generally associated with *in utero* lethality in non-mosaic males and most of the currently reported male patients show mosaicism due to *de novo* post-zygotic mutations in the *PORCN* gene. There is only one previous report of a surviving male with an inherited mutation in the *PORCN* gene. Here, we report two male siblings with multiple malformations including skeletal, ocular and renal defects overlapping with FDH. A novel *PORCN* mutation (p.Ser250Phe) was identified in a non-mosaic, hemizygous state in one of the siblings who survived to 8 years of age. The mother is a heterozygous carrier, has a random X-inactivation pattern and is asymptomatic. Findings unusual for FDH include dysplastic clavicles and bilateral Tessier IV facial clefts. This is the second case report of a non-mosaic *PORCN* mutation in a male individual with multiple congenital anomalies. While the pathogenicity of this mutation remains to be further investigated, the survival of a male with a non-mosaic mutation in *PORCN* is suggestive of a functionally mild mutation leading to an X-linked recessive mode of inheritance.

## 1 Introduction

Focal dermal hypoplasia (FDH) was first described in three females by Dr. Robert W. Goltz and Dr. Robert J. Gorlin [1]. Also known as Goltz syndrome or Goltz-Gorlin syndrome, it has since been established to be an X-linked dominant disorder caused by mutations in the *PORCN* gene [2], [3], [4]. This syndrome is more common in females, as germline mutations of this gene generally result in embryonic lethality in hemizygous males. A small number of mosaic male cases have been reported [5], [6], [7], [8], [9], caused either due to *de novo* post-zygotic mutations occurring during development or as a result of a XXY gonosome constitution.

Individuals with FDH have linear areas of thin, hyperpigmented skin due to hypoplasia of the underlying connective tissue. The collagen-containing connective tissue has been shown in some cases to be replaced by fat-containing cells and the affected individuals often have yellow nodules of fat deposition on affected areas of the skin [1], [10], [11]. Individuals with FDH also exhibit various abnormalities in tissues of ectodermal and mesodermal origin such as sparse hair, brittle nails, dental malformations, ocular defects, cleft lip and cleft palate [10], [12]. Digits of the hands and toes often show syndactyly, oligodactyly or polydactyly. Other common skeletal defects include short stature, missing long bones in the appendages and osteopathia striata [13].

Here we report a non-mosaic male with multiple congenital anomalies overlapping with FDH and a germ-line mutation in the *PORCN* gene.

## 2 Materials and methods

### 2.1 Clinical study

The family was enrolled in a study approved by the Institutional Review Board at Baylor College of Medicine. Informed consent was obtained from the family.

### 2.2 Whole exome sequencing

The exome was captured on Nimblegen's SeqCap EZ V2.0 library and sequencing was conducted on Illumina HiSeq 2000. Sequence reads were aligned as described previously [14]. Sequencing achieved over 90% of targeted bases at a minimum of 20 × coverage. Variants were called, annotated and filtered as described previously [14]. Gene candidates were then assessed using databases such as dbNSFP which assesses the functional impact and the conservation of the mutations [15], Swiss-Prot for the function of the proteins, Nextprot for the expression pattern, MGI and OMIM for the phenotypes in mice and humans, and finally Genedistiller2 for a combination of some of the above databases.

### 2.3 Sanger sequencing

For validation of the whole-exome sequencing data, DNA was amplified by polymerase chain reaction (PCR) using primers designed to amplify the mutated exon (forward: GGGTATCATGTTGGGACCTG, reverse: gaatgtatgaaAGGGCCTGG). Genomic DNA from the affected child, the child's mother, and father were used for the sequencing. The amplicons were then sequenced using standard Sanger DNA sequencing at Beckman Coulter Genomics.

### 2.4 X-chromosome inactivation studies

The X-inactivation study was performed based on the protocol described by Allen et al. with modification [16]. Briefly, 100 ng of genomic leukocyte DNA was digested with and without the methylation-sensitive restriction enzyme *Hpa*II (New England Biolabs). PCR primers flanking the androgen receptor CAG_n_ repeat region were designed as follows: 5′ACCAGGTAGCCTGTGGGGCCTCTACGATGGGC3′ (forward) and 5′CCAGAGCGTGCGCGAAGTGATCCAGAACCCGG3′ (reverse), and 5 ng DNA from each sample was subjected to PCR amplification. PCR products were separated on an ABI 3770 Analyzer and analyzed with GeneMapper software. The X-inactivation percentage was calculated as described by Sharp et al. [17]. Inactivation ratios greater than 80:20 are determined as skewed X-chromosome inactivation.

## 3 Results

### 3.1 Clinical description

The parents are African-American and the 23-year-old mother had four pregnancies, including one elective and one spontaneous abortion. The mother did not exhibit any sign of FDH such as skin, limb, or ophthalmological anomalies. The first child had intrauterine growth retardation and was born prematurely at 29 weeks of gestation. He had multiple respiratory and renal problems, including severe respiratory distress syndrome, chronic lung disease, pulmonary hypertension, pneumothorax, diaphragmatic eventration, dysplastic kidneys, hydronephrosis and renal failure. Skeletal defects included a narrow face, midface hypoplasia, a broad nasal bridge, a small mandible, syndactyly of the toes bilaterally involving the third and fourth digits, as well as pseudoarthrosis of the right clavicle. Preauricular skin tags and mild microphthalmia were noted. This child required invasive ventilation using nitric oxide. Severe pulmonary hypertension and respiratory failure caused the death of this child at 42 days.

**Fig. 1:**
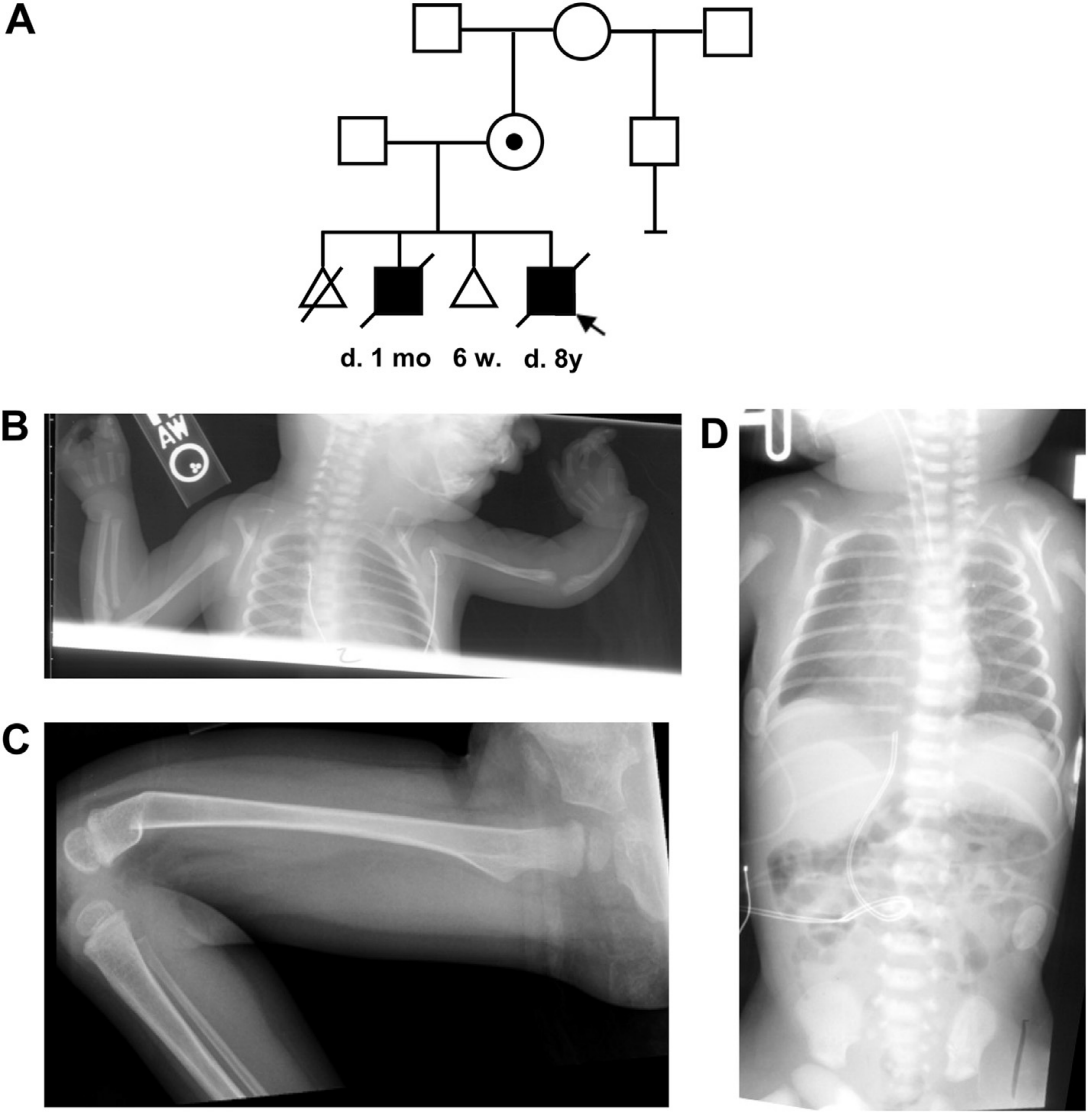
(A) Pedigree of the family described. (B) Radiographs showing the clavicular, radial and digital anomalies. (C) Radiograph showing the femoral fracture. (D) Radiograph showing a different view of the clavicular anomalies.

The second child was delivered at 35 weeks of gestation by Cesarean section due to intrauterine growth retardation and oligohydramnios. At birth, he weighed 1900 g. Facial clefts and polycystic kidneys were prenatally detected. He had a bicuspid aortic valve and pulmonary hypertension. He had relatively fair but intact skin and hypoplastic nipples that were widely spaced. His ears had underfolded helices, were low set and posteriorly rotated. Head ultrasound and hearing screen were normal. He had multicystic dysplastic kidneys and renal failure. Left cryptorchidism was also noted. Ocular defects including bilateral microphthalmia, microcornea, and a large fundus coloboma involving the optic nerve. He had bilateral clefts of the lip and cleft palate that extend to the palpebral fissures bilaterally (Tessier IV cleft). The right hand showed ectrodactyly while the left hand had syndactyly with a total of three digits. The left forearm was markedly shortened and radially angulated at the wrist. X-rays revealed bilateral hypoplasia of the radius and short ulnae (Fig. 1). The right foot had 1–2 and 3–4 syndactyly while the left foot showed mild 3–4 syndactyly. The child had hypoplastic nails. A chest X-ray showed presence of only 11 pairs of ribs. The clavicles were severely dysplastic bilaterally, the shoulders could be approximated anteriorly and the left clavicle was not palpable.

### 3.2 Mutation identification and X-inactivation studies

**Fig. 2:**
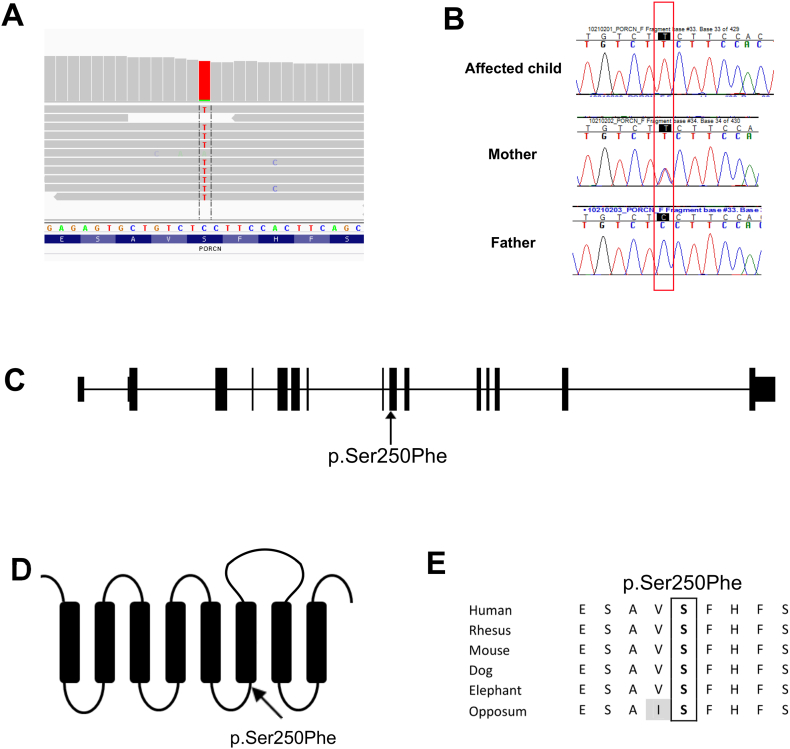
Representation of the mutation on (A) the exome sequencing alignment, (B) the Sanger sequencing results of the proband and parents, (C) the genomic context, (D) the protein diagram and (E) the amino acid conservation alignment in mammals.

Because of the recurrence in males, the absence of skin findings and the unusual features (facial clefting and almost absent clavicles), we conducted whole exome sequencing to investigate the molecular basis of disease in this family. After whole exome sequencing, variants in genes associated with skeletal dysplasias in humans or mice were considered. The *PORCN* gene mutation was identified as the most likely deleterious mutation based on his features that overlapped with the phenotype of FDH (Table 1). The mutation (c.749C > T, NM_203475.1) causes the amino acid serine at position 250 to be replaced by phenylalanine. This change was predicted to be pathogenic by 4/4 prediction programs (SIFT, Polyphen2, LRT, MutationTaster) and the position is highly conserved according to PhyloP [18]. This amino acid and nucleotide position is conserved across species even down to *C. elegans* (Fig. 2). The paper by Lombardi et al., as well as the database created by the same group, lists known mutations in the *PORCN* gene [19]. However, neither the paper, nor the associated database lists this mutation. The mutation was not found in the exome variant server (Version ESP5400, accessed December 11, 2012 which includes over 3000 alleles from African American individuals). Sanger sequencing confirmed the nucleotide change in the proband and showed that his mother is a heterozygous carrier for the same mutation (Fig. 2). X-inactivation studies showed that the mother has an A:B allele ratio of 51:49, consistent with a random X-inactivation pattern. Additional family members were not available for molecular studies.

**Table 1:** Clinical features seen in the male patients and their reported incidence in FDH patients (N/A = not available).

	Male patient 1	Male patient 2	Reported incidence in FDH	Reported incidence in Lenz microphthalmia syndrome
Typical skin findings (fat herniation, aplasia, hyperpigmentation or poikilodermia)	−	−	Frequent	Not reported
Microphthalmia and other ocular defects	+	+	Frequent	Frequent
Cleft lip and cleft palate	−	+	Occasional	Occasional
Syndactyly	+	+	Frequent	Frequent
Ectrodactyly	−	+	Frequent	Not reported
Dysplastic nails	N/A	+	Frequent	Not reported
Osteopathia striata	N/A	+	Frequent	Not reported
Clavicular dysplasia	+	+	Occasional	Frequent
Costovertebral segmentation abnormalities	−	+	Frequent	Not reported
Diaphragmatic hernia	+	−	Occasional	Not reported
Cardiac anomalies and pulmonary hypertension	+	+	Occasional	Occasional
Renal anomalies	+	+	Occasional	Frequent

## 4 Discussion

Even though the proband lacked the characteristic feature of hypoplasia of the skin, he possessed other key features of FDH including microphthalmia and skeletal anomalies such as syndactyly, brachydactyly, absence of a rib pair and radial hypoplasia. Clavicular dysplasia has been described in a few individuals with FDH [20], [21]. Osteopenia, fractures, and osteopathia stratia-like lesions seen in this affected individual are also frequently seen in other individuals with focal dermal hypoplasia [10], [22], [23], [24].

Facial clefts are occasionally reported in FDH, and very few defined genetic syndromes are known to lead to facial clefts [25], [13]. Renal abnormalities, though rare, have been previously reported in FDH, including multicystic kidneys [13], [26]. Nipple hypoplasia is also a feature of FDH [27]. Finally, diaphragmatic hernia and pulmonary hypertension are also seen in focal dermal hypoplasia, albeit rarely [10], [28], [29].

Another condition to consider in this family is Lenz microphthalmia. Indeed, the microphthalmia, syndactyly, clavicular dysplasia, cardiac and renal anomalies could be compatible with Lenz microphthalmia [30]. The individuals described here did not have the dental anomalies, duplicated thumbs and webbed neck often seen in Lenz microphthalmia (Table 1). They also had ectrodactyly, dysplastic nails, lesions reminiscent of osteopathia striata, costovertebral segmentation abnormalities, and diaphragmatic hernia which have never been reported in Lenz microphthalmia but are seen in FDH. Moreover, BCOR was covered by at least 100 reads for all coding nucleotides and no mutations were identified by Next-Generation sequencing. Also, since this gene is on the X chromosome, a partial or complete gene deletion would have been noted on the exome alignment in the male individual described here.

The mother of the proband in our study, despite being a carrier, shows no clinical symptoms of FDH and showed a random X-inactivation pattern. However, this disparity could be because of differences in X-inactivation between tissue types. Alternatively, it is also possible that she has a *de novo* mutation in the *PORCN* gene and displays mosaicism.

A recently published whole-exome sequencing study has also reported two non-mosaic males with clinical symptoms overlapping FDH with an inherited mutation in the PORCN gene [31]. Both male patients in this study had a c.407G > A mutation change resulting in a Glycine to Aspartate amino acid change in the protein which is different from the variant and amino acid change that we report here. There were some clinical differences between the patients for example neither of the male patients in our study had spina bifida while neither patient reported in the previous study showed syndactyly or ectrodactyly of the fingers and toes. Both male patients in the previous study had normal weight at birth and showed no clefting of the lip or palate. However, there were several similarities as well. Interestingly, none of the male patients reported in either study had the characteristic skin findings that are associated with FDH. Diaphragmatic hernia and hydronephrosis of the kidney were observed in 1 male patient in each study. Anomalies in the radial bone were also seen in 1 patient in each study. Both studies also reported microphthalmia and other ocular defects such as coloboma of the retina. The differences in clinical presentation can likely be attributed to the different variant observed in the patients. The similarities in clinical presentation lend further evidence that inherited mutations in the *PORCN* gene can lead to surviving males with clinical symptoms similar to those in FDH.

## 5 Conclusion

This is the second report of a male child with congenital anomalies overlapping FDH, associated with a non-mosaic mutation in *PORCN* gene. Most previous reports of surviving males with FDH have described *de novo* somatic mutations acquired during embryonic development leading to a mosaicism and father-to-daughter transmission. The survival of a male child with a non-mosaic germ-line mutation in *PORCN* inherited from the mother is suggestive of a mutation only mildly affecting the protein and leading to an X-linked recessive mode of inheritance, with asymptomatic heterozygous females and severely affected hemizygous males.

## Competing interests

The authors have no conflicts of interest to declare.
